# Influence of Nitrogen Nutrition on Fatty Acids in Oilseed Rape (*Brassica napus* L.)

**DOI:** 10.3390/plants11010044

**Published:** 2021-12-24

**Authors:** Alexandra Zapletalová, Ladislav Ducsay, Ladislav Varga, Jakub Sitkey, Soňa Javoreková, Peter Hozlár

**Affiliations:** 1 Institute of Agronomical Sciences, Faculty of Agrobiology and Food Resources, Slovak University of Agriculture, 94901 Nitra, Slovakia; alexandra.zapletalova@uniag.sk (A.Z.); ladislav.varga@uniag.sk (L.V.); xsitkey@uniag.sk (J.S.); 2 Institute of Biotechnology, Faculty of Biotechnology and Food Sciences, Slovak University of Agriculture, 94901 Nitra, Slovakia; sona.javorekova@uniag.sk; 3 National Agricultural and Food Center, Research Institute of Plant Production, Research and Breeding Station Vígľaš Pstruša, 96212 Detva, Slovakia; peter.hozlar@nppc.sk

**Keywords:** fertilizer DAN 27, qualitative parameters, linoleic acid, linolenic acid, oleic acid

## Abstract

The aim of the study was to evaluate the effect of nitrogen nutrition on the content of fatty acids and selected qualitative parameters (nitrogenous substances, ash, crude fiber) in winter oilseed rape (*Brassica napus* L.). The experiment was carried out at the Vígľaš—Pstruša Research and Breeding Station in 2008–2009 and 2009–2010 by complete block design with four repetitions. Nitrogen fertilization was applied at four levels, plus an untreated control (after agrochemical soil analysis) by DAN 27 (Dolomite Ammonium Nitrate): 100, 120, 140, and 160 kg/ha N. Application date was in BBCH scale phase 59–60. The fatty acid contents (MUFA—monosaturated fatty acids; PUFA—polyunsaturated fatty acids) were determined by gas chromatography in the extracted fat, which is determined by extraction method. Within the result evaluation, statistically significant increases in the contents of linoleic and linolenic acids were recorded in all variants treated by nitrogen fertilizer, which is positive in terms of the use of rapeseed oil for food and energy purposes. The statistically significant decrease of oleic acid after the application of nitrogen fertilizers is negative for industry use of rapeseed oil.

## 1. Introduction

Oilseed rape (*Brassica napus* L.) is one of the most important oilseed crops in the Slovak Republic. Canada (19 million metric tons), the EU (16.83 million metric tons), China (13.1 million metric tons), and India (7.7 million metric tons) are in the leading positions in rapeseed production [1].

Oilseed rape disposes with a very good adaptability to external environmental conditions and its main use is concentrated in the food industry. For human nutrition, oils such as refined edible oil with quality 00, cold-pressed edible oil, and oil of HOLLi quality with a very low content of α-linolenic acid of 3% are used. Refined and cold-pressed rapeseed oils are valuable due to their high α-linolenic acid content, about 10%, low saturated fatty acid content, about 6%, and an optimal n-6:n-3 ratio of 2:1 [2]. The percentage of major fatty acids in the oil of rapeseed cultivars are on average 59–68%, linoleic acid 17–21%, and linolenic acid 7.8–10% [3]. The value of rapeseed as a source of vegetable oils and proteins is possible to improve by increasing the oil content, adjusting the fatty acid composition, and by reducing antinutritional substance content, especially fiber and glucosinolates in rapeseed flour [4]. Oilseed rape is also used in fodder (animal nutrition), in oil chemistry (production of special substances such as glycerin, amines, esters, soaps, paints, varnishes), and for energy purposes (biodiesel, MERO) [5,6].

Several experiments confirm the impact of individual interventions on amount and seed quality in rapeseed cultivation technology [7]. Improvement in cultivation technology leads to biological progress, which is associated with intensification of fertilization and pesticide application, which negatively affects the final consumer. Therefore, it is necessary to improve cultivation systems so that not only the farmer but also the consumer will be satisfied [8,9].

The often-discussed theme of nutrition and fertilization is not only oriented on oilseed rape, but also on other agricultural crops. Harmonious nutrition, especially used on nutritionally poor soils, can affect crop yields and crop quality positively [10,11]. Despite the need to apply macro- and micronutrients for proper plant growth and development, nitrogen and sulfur appear to be the most restrictive nutrients [12]. Research has shown that not always increasing the nitrogenous substance amount, which has a positive effect on crude protein, has affected the oil content or caused its decrease [13,14,15]. The chemical analyses confirmed non-significant effects of different doses of nitrogen on the rapeseed oil content and its fatty acids (palmitic acid, stearic acid, linoleic acid, linolenic acid, arachidic acid, and erucic acid). A very low erucic acid content (0.1–0.9%) has occurred [16].

The aim of this study was to evaluate the effect of nitrogenous nutrition on higher fatty acids amount and selected qualitative parameters in seeds of winter *Brassica napus* L.

## 2. Results

### 2.1. Higher Fatty Acids Contents

The statistically significant effect of year was found in contents of oleic, linoleic, linolenic, behenic, and erucic acid. Statistically significant increasing was achieved in linoleic, linolenic, behenic acid, and decreasing in oleic acid in 2009–2010 (Table 1A).

As an average of years 2008–2009 and 2009–2010, statistically significant differences in fatty acids contents between nitrogen treatments were recorded (Table 1A). After nitrogen nutrition, the statistically significant increases were reported in average percentage contents of linoleic acid on all treated variants in comparison with untreated variants. The average highest increase (18.81 % of total fatty acid content) was found on the variant with treatment to 140 kg/ha N compared with control variant (17.76%). The statistically significantly highest content of linoleic acid (19.57 ± 0.04%) was achieved on treated variant to 140 kg/ha N in comparison with untreated variant in 2009–2010 (Table 1A).

After nitrogen treatment, statistically significant increases of linolenic acid were found on all variants in comparison with the control variant (Table 1B). On average, the statistically significant highest increase (8.64%) of linolenic acid was found on variant fertilized to 160 kg/ha N in comparison with untreated variant (8.16%). The statistically significantly highest content of linolenic acid (9.50 ± 0.05%) was found on fertilized variant to 140 kg/ha N in comparison with untreated variant in 2009–2010.

On average, statistically significant decreases of oleic acid after nitrogen fertilizers were found in comparison with untreated control. The statistically significantly greatest decrease, on average 64.55%, was achieved on variant with treatment to 120 kg/ha N in comparison with untreated control (66.62%). Statistically significantly lowest content of oleic acid (62.81 ± 0.34%) was achieved on treated variant to 120 kg/ha N in comparison with control variant in 2009–2010.

In evaluation of other fatty acids average values (palmitic, palmitoleic, stearic, arachic, erucic, and lignoceric) the effect of nitrogen fertilizers was found statistically non-significant in comparison with the untreated control variant (Table 1A,B).

### 2.2. Qualitative Parameters

Within evaluation of qualitative parameters (nitrogenous substances, ash, crude fiber) statistically significant differences were found in average of years 2008–2009 and 2009–2010. Statistically significant increasing of nitrogenous substances and ash were confirmed in oilseed rape seeds on all variants with nitrogen treatments. Statistically significant decrease (16.35%) of crude fiber content was found on variants treated to 140 kg/ha N in comparison with untreated variants.

Statistically significant increasing of nitrogenous substance content in seeds of oilseed rape was found on treated variants in comparison with control variant in year 2008. The nitrogen influence on ash content was statistically non-significant. Within evaluation of crude fiber, statistically significant difference was confirmed only between nitrogen treated variants fertilized to 120 kg/ha N and 160 kg/kg N (Table 2).

Statistically significant decrease of crude fiber was found after nitrogen nutrition in year 2009. Decrease of 26.56% was found in comparison with untreated variant. The effect of application of nitrogen fertilizers was statistically non-significant regarding the content of nitrogenous substances and ash in seeds of oilseed rape (Table 2).

## 3. Discussion

Oilseed rape can be also grown to produce biodiesel [8,17,18]. Biodiesel disposes with several other benefits (low sulfur content, net CO_2_ emissions compared with conventional type of diesel, non-toxicity, and biodegradability) [19].

Fatty acids can be divided into classes of saturated, monosaturated (MUFA), and polyunsaturated (PUFA) fatty acids. Whereas biodiesel is made from fatty acid esters, it is important to know the ester profiles of biodiesel. Ideally, the vegetable oil used to produce of biodiesel has a relatively higher percentage of MUFA than PUFA. In most raw materials for the biodiesel production from vegetable oils, the main occurring fatty acids are oleic, linoleic, palmitic, and stearic acids, and oleic is the dominant acid [20,21,22]. Oleic and linoleic acids create a part of the unsaturated fatty acids of a biodiesel sample, meaning fatty acids with one or more double bonds in their molecular structure. From discussions of poor oxidative stability of diesel with high content of PUFA (linoleic), for good oxidative stability, the diesel should contain more saturated fatty acids [20,23]. Results showed that the fatty acid composition is an integral part of biodiesel production because it has a major impact on the physico-chemical fuel properties of biodiesel. The results of the study showed that unsaturated fatty acids predominate in the evaluated samples, which are desirable for the correct properties of biodiesel. The main fatty acids in biodiesel fuels range from 69.00% to 77.81% of unsaturated fatty acids. The total results concluded that the fatty acid composition affects the fuel properties of the studied biodiesel [24].

For the food industry, rapeseed oil is considered as one of the healthiest vegetable oils for its fatty acid composition. It is characterized by a low content of saturated fatty acids (6–7%), a high content of MUFA, mainly oleic acid (58–62%) and PUFA represented by α-linolenic acid (n-3) 8–12% and linoleic acid (n-6) about 20% [25,26]. Fat within foods contains a mixture of different types of fatty acids (saturated, monounsaturated, polyunsaturated). Butter, coconut oil, lard, dripping, ghee, and palm oil contain high amounts of saturated fat whereas rapeseed, olive, and peanut oils are high in monounsaturated fat. High intakes of saturated fat have been shown to raise levels of low-density lipoprotein (LDL, or ‘bad’) cholesterol in the blood and high LDL cholesterol increases the risk of developing heart disease and stroke [27]. Here is also some evidence to suggest a link between saturated fatty acids and many human diseases (cancer, insulin resistance, metabolic syndrome, obesity) [28]. It is therefore recommended that saturated fat in the diet is reduced and replaced with small amounts of unsaturated fats and oils such as olive and rapeseed oil as this has been found to reduce blood cholesterol levels [29].

The nitrogen application in the production process of a selected crop for the producing of biofuels or biodiesel, has a major impact not only on the overall profitability of raw material systems for biofuel production but also on the environment [18,30]. For great interest of farmers in various oil crop alternatives, different levels of nitrogen fertilization (0, 50, 100, 150 kg/ha N) per oil content and fatty acid composition were observed in field trials in the southeastern United States [19]. Nitrogen fertilization to 150 kg/ha increased the linolenic acid content. Our results confirmed that the effect of nitrogen nutrition was an increase in linoleic and linolenic acid not only on average in 2008–2009 and 2009–2010 but also within individual years. On average in 2008–2009 and 2009–2010, as well as in 2008 and 2009, decreases in oleic acid contents were found in all variants treated by nitrogen fertilizers. From the achieved results, it follows that the increase in linoleic and linolenic acid (PUFA) in rapeseed are suitable for both uses. However, the decrease in oleic acid (MUFA) content in the seed after nitrogen nutrition can be the aim of other studies and experiments because nitrogen is one of the yield-creating elements. An interesting fact remains the possible relation with weather conditions of cultivation year, when during the butonization (beginning of flowering in April) the temperatures were strongly above the normal and the total precipitation was strongly below the normal.

## 4. Material and Methods

### 4.1. Research Material

Field small plot experiments were carried out at the Research and Breeding Station (RBS) Vígľaš—Pstruša in years 2008–2010. The experiments were aimed to determine the effect of nitrogen nutrition on fatty acid content in seeds of oilseed rape and selected qualitative parameters. Baldur, the hybrid of oilseed rape (*Brassica napus* L.), was used in the experiment. The experimental area RBS Vígľaš–Pstruša is characterized like a potato and wheat production area (III-C2) with a height of 375 m above the sea level. The soil type is clay loam Luvisol Pseudogley, with topsoil depth 0.3 m. Soil reaction is 5.57. Soil agrochemical analysis of experimental field before experimental establishment with winter oilseed rape to the depth 0.3 m in experimental years 2008–2009 and 2009–2010 is described in Table 3.

Fertilization was based on untreated control (without N fertilizers), and four variants with applying DAN 27 in doses to 100 120, 14, and 160 kg/ha N in BBCH 59–60. DAN 27 is a nitrogen fertilizer containing 27% of nitrogen and 4% of MgO. It is a mixture of ammonium nitrate with finely ground dolomite in the form of whitish to light brown granules sized 2–5 mm.

### 4.2. Research Methods

The experiment used split plot design with randomized complete block design in four repetitions. The plot size was 10 m^2^. The average annual temperature in season (IV–IX) is 14 °C. The snow cover lasts 60 days of the year, what was not the rule in recent years. The average annual precipitations are 666 mm. More detailed characteristics of weather conditions in experimental years are given in Table 4 and Table 5.

Determination of crude fiber was realized in the Dosi fiber machine. The samples were given in the machine under glass tubes. Tubes were filled by hot H_2_SO_4_ 0.128 M with temperature 70 °C in 30 min. Afterwards, they were extracted with hot water 3×. Tubes were filled by hot 0.23 M NaOH in 30 min and after were extracted with hot water 3×. The samples were poured over with acetone 3×, and they dried. Next, the samples were dried in an oven for 2 h at 150 °C and weighed. Then they were left for 2 h at 500 °C in a muffle furnace. Afterwards, they were weighed and fiber percentage was calculated.

Determination of ash was based on weighing the samples and were left in a muffle furnace for 5 h at 450 °C. Afterwards, the samples were weighed and ash percentage was calculated.

Determination of nitrogenous substances was based on weighing the samples, filled by 10 mL H_2_SO_4_, mineralized, and titrated by 0.1 M H_2_SO_4_ and percentage of nitrogenous substances was calculated.

Determination of fatty acids—for the characteristic of lipid fraction triglycerides to glycerol and free fatty acids were hydrolyzed. Fatty acids were then derivated to methylesters. After their preparation they were separated based on carbon number and level of unsaturation by using gas chromatography fitted with a flame-ionization detector (FID). For the identification column 37 components mixture (Supelco 47885-U) was used. Standard solution was diluted with 10 mL of hexane with 1 mL supplementation of 2 N potassium hydroxide in methanol. Analytic tube was heated for 30 s at 60 °C in a water bath. After 1 min, 2 mL of 1 N hydrochloric acid was added. The top layer was transferred in an amount of 2 mL to an autosampler vial with ninhydrin (Na_2_SO_4_). Injection of samples was performed by injection autosampler Agilent. The content of fatty acids on machine Agilent 6890A GC (Agilent Technologies, 23 Mill Street Arcade, NY 14009, USA) as a percentage in crude fat was determined [31].

### 4.3. Statistical Analysis

The achieved experimental results were statistically evaluated by standard methods using the Statgraphics plus 5.1 statistical software (Rockville, Maryland, USA). A multifactor ANOVA and one-way ANOVA models were used for the individual treatment comparison at *p =* 0.05, with separation of the means by the *LSD* multiple-range test.

## 5. Conclusions

The content of fatty acids, observed in this study, was affected by nitrogen fertilizer application. Increase of PUFA in achieved oil, linolic and linolenic acids, is positive for food and energy purposes. Decrease of oleic acids (MUFA), which are important for food industry, was caused by nitrogen fertilizer application. This is important knowledge for agricultural practice.

The contents of nitrogenous substances and ash increased by application of nitrogen fertilizers, but in comparison the crude fiber content decreased in average of the years 2008–2009 and 2009–2010.

## Figures and Tables

**Table 1:** Influence of year and nitrogenous nutrition on fatty acids (% of total fatty acids content).

(A)															
Variant	Palmitic Acid			Palmitoleic Acid			Stearic Acid			Oleic Acid			Linoleic Acid		
	2008–2009	2009–2010	Mean	2008–2009	2009–2010	Mean	2008–2009	2009–2010	Mean	2008–2009	2009–2010	Mean	2008–2009	2009–2010	Mean
Variant 0 kg/ha N	4.55 ± 0.12 ^a^	4.39 ± 0.04 ^a^	4.47 ^a^	0.28 ± 0.02 ^a^	0.27 ± 0.02 ^ab^	0.27 ^ab^	1.79 ± 0.02 ^b^	1.82 ± 0.01 ^a^	1.81 ^a^	67.27 ± 0,31 ^c^	65.97 ± 2.35 ^b^	66.62 ^b^	17.31 ± 0.02 ^a^	18.21 ± 1.11 ^a^	17.76 ^a^
Fertilized to 100 kg/ha N	4.49 ± 0.01 ^a^	4.42 ± 0.00 ^a^	4.46 ^a^	0.28 ± 0.01 ^a^	0.29 ± 0.01 ^b^	0.28 ^b^	1.75 ± 0.01 ^ab^	1.95 ± 0.01 ^c^	1.85 ^a^	65.89 ± 0.08 ^a^	63.60 ± 0.02 ^ab^	64.74 ^a^	18.06 ± 0.04 ^b^	19.33 ± 0.010 ^ab^	18.70 ^b^
Fertilized to 120 kg/ha N	4.48 ± 0.01 ^a^	4.54 ± 0.16 ^ab^	4.51 ^a^	0.27 ± 0.02 ^a^	0.23 ± 0.04 ^a^	0.25 ^a^	1.75 ± 0.01 ^ab^	1.88 ± 0.01 ^b^	1.82 ^a^	66.28 ± 0.03 ^ab^	62.81 ± 0.34 ^a^	64.55 ^a^	18.02 ± 0.03 ^b^	18.76 ± 0.04 ^ab^	18.39 ^b^
Fertilized to 140 kg/ha N	4.55 ± 0.01 ^a^	4.56 ± 0.02 ^ab^	4.55 ^a^	0.26 ± 0.01 ^a^	0.27 ± 0.00 ^ab^	0.26 ^ab^	1.71 ± 0.05 ^a^	1.86 ± 0.01 ^b^	1.78 ^a^	66.52 ± 0.04 ^b^	63.13 ± 0.06 ^a^	64.82 ^a^	18.06 ± 0.06 ^b^	19.57 ± 0.04 ^b^	18.81 ^b^
Fertilized to 160 kg/ha N	4.51 ± 0.13 ^a^	4.66 ± 0.01 ^b^	4.58 ^a^	0.28 ± 0.01 ^a^	0.27 ± 0.00 ^ab^	0.27 ^ab^	1.74 ± 0.04 ^ab^	1.88 ± 0.01 ^b^	1.81 ^a^	65.88 ± 0.14 ^a^	63.49 ± 0.01 ^ab^	64.68 ^a^	18.20 ± 0.15 ^b^	19.36 ± 0.06 ^ab^	18.78 ^b^
Standard error			0.0460			0.0094			0.0210			0.4080			0.1691
Year	4.51 ^a^	4.52 ^a^		0.27 ^a^	0.26 ^a^		1.75 ^a^	1.88 ^a^		66.37 ^b^	63.80 ^a^		17.93 ^a^	19.04 ^b^	
Standard error			0.029			0.006			0.013			0.013			0.260
**(B)**															
**Variant**	**Linolenic Acid**			**Arachic Acid**			**Behenic Acid**			**Erucic Acid**			**Lignoceric Acid**		
	**2008–2009**	**2009–2010**	**Mean**	**2008–2009**	**2009–2010**	**Mean**	**2008–2009**	**2009–2010**	**Mean**	**2008–2009**	**2009–2010**	**Mean**	**2008–2009**	**2009–2010**	**Mean**
Variant 0 kg/ha N	7.16 ± 0.01 ^a^	9.16 ± 0.01 ^a^	8.16 ^a^	0.53 ± 0.08 ^a^	0.49 ± 0.01 ^a^	0.51 ^a^	0.30 ± 0.05 ^a^	0.33 ± 0.01 ^a^	0.31 ^a^	0.21 ± 0.02 ^ab^	0.11 ± 0.08 ^a^	0.16 ^ab^	0.20 ± 0.03 ^ab^	0.14 ± 0.01 ^a^	0.17 ^ab^
Fertilized to 100 kg/ha N	7.96 ± 0.03 ^c^	9.27 ± 0.01 ^b^	8.61 ^b^	0.60 ± 0.01 ^a^	0.55 ± 0.01 ^b^	0.58 ^a^	0.35 ± 0.01 ^a^	0.40 ± 0.01 ^a^	0.37 ^b^	0.27 ± 0.03 ^bc^	0.07 ± 0.05 ^a^	0.17 ^ab^	0.36 ± 0.16 ^b^	0.15 ± 0.02 ^ab^	0.25 ^ab^
Fertilized to 120 kg/ha N	7.95 ± 0.01 ^c^	9.28 ± 0.01 ^b^	8.61 ^b^	0.50 ± 0.01 ^a^	0.54 ± 0.04 ^ab^	0.52 ^ab^	0.31 ± 0.01 ^a^	0.40 ± 0.07 ^a^	0.35 ^ab^	0.23 ± 0.01 ^b^	0.10 ± 0.07 ^a^	0.16 ^ab^	0.24 ± 0.02 ^ab^	0.38 ± 0.08 ^c^	0.31 ^b^
Fertilized to 140 kg/ha N	7.76 ± 0.04 ^b^	9.50 ± 0.05 ^c^	8.63 ^b^	0.56 ± 0.07 ^a^	0.52 ± 0.01 ^ab^	0.54 ^ab^	0.33 ± 0.02 ^a^	0.36 ± 0.04 ^a^	0.34 ^ab^	0.13 ± 0.01 ^a^	0.12 ± 0.01 ^a^	0.12 ^a^	0.13 ± 0.01 ^a^	0.15 ± 0.01 ^ab^	0.14 ^a^
Fertilized to 160 kg/ha N	8.01 ± 0.10 ^c^	9.27 ± 0.02 ^b^	8.64 ^b^	0.57 ± 0.01 ^a^	0.53 ± 0.01 ^ab^	0.55 ^ab^	0.30 ± 0.04 ^a^	0.32 ± 0.04 ^a^	0.31 ^a^	0.37 ± 0.08 ^c^	0.11 ± 0.03 ^a^	0.24 ^b^	0.16 ± 0.02 ^a^	0.24 ± 0.01 ^b^	0.20 ^ab^
Standard error			0.0921			0.0192			0.0193			0.0333			0.0461
Year	7.77 ^a^	9.29 ^b^		0.55 ^a^	0.53 ^a^		0.31 ^a^	0.36 ^b^		0.24 ^b^	0.10 ^a^		0.22 ^a^	0.21 ^a^	
Standard error			0.107			0.058			0.012			0.012			0.020

**Table 2:** Influence of year and nitrogenous nutrition on qualitative parameters (%).

Variant	Nitrogenous Substances (%)			Ash (%)			Crude Fiber (%)		
	2008–2009	2009–2010	Mean	2008–2009	2009–2010	Mean	2008–2009	2009–2010	Mean
Variant 0 kg/ha N	183.14 ± 7.18 ^a^	232.64 ± 10.57 ^a^	207.89 ^a^	42.65 ± 3.05 ^a^	41.74 ± 1.89 ^a^	42.16 ^a^	222.53 ± 8.73 ^ab^	216.64 ± 9.84 ^b^	219.59 ^b^
Fertilized to 100 kg/ha N	236.30 ± 10.39 ^b^	254.62 ± 12.02 ^a^	245.45 ^b^	44.56 ± 1.96 ^a^	43.17 ± 2.04 ^a^	43.86 ^bc^	229.09 ± 10.08 ^ab^	191.83 ± 9.05 ^a^	210.46 ^ab^
Fertilized to 120 kg/ha N	238.94 ± 10.68 ^b^	252.54 ± 11.31 ^a^	245.74 ^b^	43.99 ± 1.97 ^a^	42.84 ± 2.60 ^a^	43.42 ^ab^	206.63 ± 9.22 ^a^	207.84 ± 9.31 ^ab^	207.07 ^ab^
Fertilized to 140 kg/ha N	236.84 ± 16.97 ^b^	250.22 ± 11.40 ^a^	243.53 ^b^	43.69 ± 1.89 ^a^	43.04 ± 3.33 ^a^	43.36 ^ab^	209.63 ± 9.03 ^a^	196.85 ± 8.97 ^ab^	203.24 ^a^
Fertilized to 160 kg/ha N	244.45 ± 10.92 ^b^	249.77 ± 8.85 ^a^	247.11 ^b^	46.10 ± 2.06 ^a^	43.29 ± 4.29 ^a^	44.69 ^c^	231.31 ± 31 ^b^	190.08 ± 6.73 ^a^	210.69 ^ab^
Standard error			4.8637			0.4113			5.3142
Year	227.93 ^a^	247.96 ^b^		44.19 ^a^	42.81 ^b^		219.77 ^a^	200.65 ^b^	
Standard error			3.0761			0.2601			0.3610

**Table 3:** Agrochemical soil analysis of experimental area.

Nan (sum of N-NH_4_^+^ a N-NO_3_^−^):	2008–2009—10.0 mg/kg soil 2009–2010—11.1 mg/kg soil
N-NH_4_^+^ (colorimetrically, Nessler’s reagent):	2008–2009—4.0 mg/kg soil 2009–2010—4.6 mg/kg soil
N-NO_3_^−^ (colorimetrically, phenol 2,4-disulfonic acid)	2008–2009—6.0 mg/kg soil 2009–2010—6.3 mg/kg soil
P—available (colorimetrically, Mehlich III):	2008–2009—96.3 mg/kg soil 2009–2010—89.6 mg/kg soil
K—available (flame photometry, Mehlich III):	2008–2009—190.0 mg/kg soil 2009–2010—206.3 mg/kg soil
Mg—available (AAS, Mehlich III):	2008—275.0 mg/kg soil 2009—298.6 mg/kg soil
Ca—available (flame photometry, Mehlich III):	2008–2009—1450 mg/kg soil 2009–2010—1965 mg/kg soil
% of humus	2008–2009—2.68 2009–2010—2.36
pH/KCl (0.2 mol/dm^3^ KCl) (pH units)	2008–2009—5.0 2009–2010—5.8

**Table 4:** Monthly means of air temperature compared with the long-term average 1961–2001.

Month	Long-Term Average 1961–2001	2007–2008		2008–2009		2009–2010	
		Temperature (°C)	Evaluation of Year	Temperature (°C)	Evaluation of Year	Temperature (°C)	Evaluation of Year
I.	−3.8	-	-	−4.4	N	−2.9	N
II.	−1.5	-	-	−1.1	N	−1.0	N
III.	2.8	-	-	3.4	N	2.5	N
IV.	8.4	-	-	11.5	SAN	9.1	N
V.	13.1	-	-	14.4	N	13.6	N
VI.	16.3	-	-	16.1	N	17.5	AN
VII.	17.8	-	-	19.8	SAN	20.6	SAN
VIII.	17.3	-	-	19.5	SAN	-	-
IX.	13.2	12.8	N	15.3	SAN	-	-
X.	8.1	10.0	AN	8.5	N	-	-
XI.	3.00	4.9	SAN	4.8	SAN	-	-
XII.	−1.6	1.5	SAN	−0.8	N	-	-
IV.–IX.	15.7	-	-	16.1	N	-	-
X.–III.	1.2	-	-	2.4	SAN	1.9	AN
I.–XII.	7.8	-	-	8.9	N	-	-

Legend: N—normal; AN—above normal; BN—below normal; SAN—strongly above normal; SBN—strongly below normal; EAN—extremely above normal.

**Table 5:** Monthly sums of precipitation compared with the long-term average 1961–2001.

Month	Long-Term Average 1961–2001	2007–2008		2008–2009		2009–2010	
		Precipitation (mm)	Evaluation of Year	Precipitation (mm)	Evaluation of Year	Precipitation (mm)	Evaluation of Year
I.	28.1	-	-	39.2	AN	43.1	AN
II.	28.5	-	-	40.2	AN	34.7	N
III.	29.8	-	-	19.4	AN	52.2	AN
IV.	46.7	-	-	11.0	SBN	55.0	N
V.	63.9	-	-	62.8	N	132.8	SAN
VI.	85.2	-	-	96.4	N	207.1	EAN
VII.	75.6	-	-	34.2	BN	100.2	AN
VIII.	61.6	-	-	35.6	N	-	-
IX.	49.5	40.9	N	37.8	N	-	-
X.	45.7	49.8	N	115.9	SAN	-	-
XI.	53.5	35.8	N	81.6	AN	-	-
XII.	41.8	83.5	SAN	101.6	SAN	-	-
IV.–IX.	63.8	-	-	46.3	N	-	-
X.–III.	37.9	-	-	49.7	SAN	71.5	EAN
I.–XII.	50.83	-	-	58.8	AN	-	-

Legend: N—normal; AN—above normal; BN—below normal; SAN—strongly above normal; SBN—strongly below normal; EAN—extremely above normal.

## Data Availability

Not applicable.
